# Supplementary material for: Diagnostic Accuracy of the Abbott BinaxNOW COVID‐19 Antigen Card Test, Puerto Rico
Source: Influenza Other Respir Viruses. 2024 Jul 25;18(7):e13305. doi: 10.1111/irv.13305 (PMC11300111; doi:10.1111/irv.13305)
Supplement: Supplementary file 1 — Figure S1. Sensitivity and specificity of BinaxNOW Antigen test compared to RT‐PCR by number of COVID‐19 symptoms (N = 1524 paired tests from 1201 participants experiencing 0 to 16 symptoms). Figure S2. Sensitivity of BinaxNOW Antigen test compared to RT‐PCR by project (COPA, SEDSS) and number of symptoms reported (N = 1526 paired tests from 1203 participants). Table S1. Definitions of diagnostic performance metrics Table S2. Comparison of BinaxNOW and RT‐PCR for initial tests and repeated tests 7–14 days later by symptom status for the initial and repeated tests (N = 368 paireda tests from 184 participants). [file IRV-18-e13305-s001.docx]

**Supplementary Materials**

**Diagnostic Accuracy of the Abbott BinaxNOW COVID-19 Antigen Card Test, Puerto Rico**


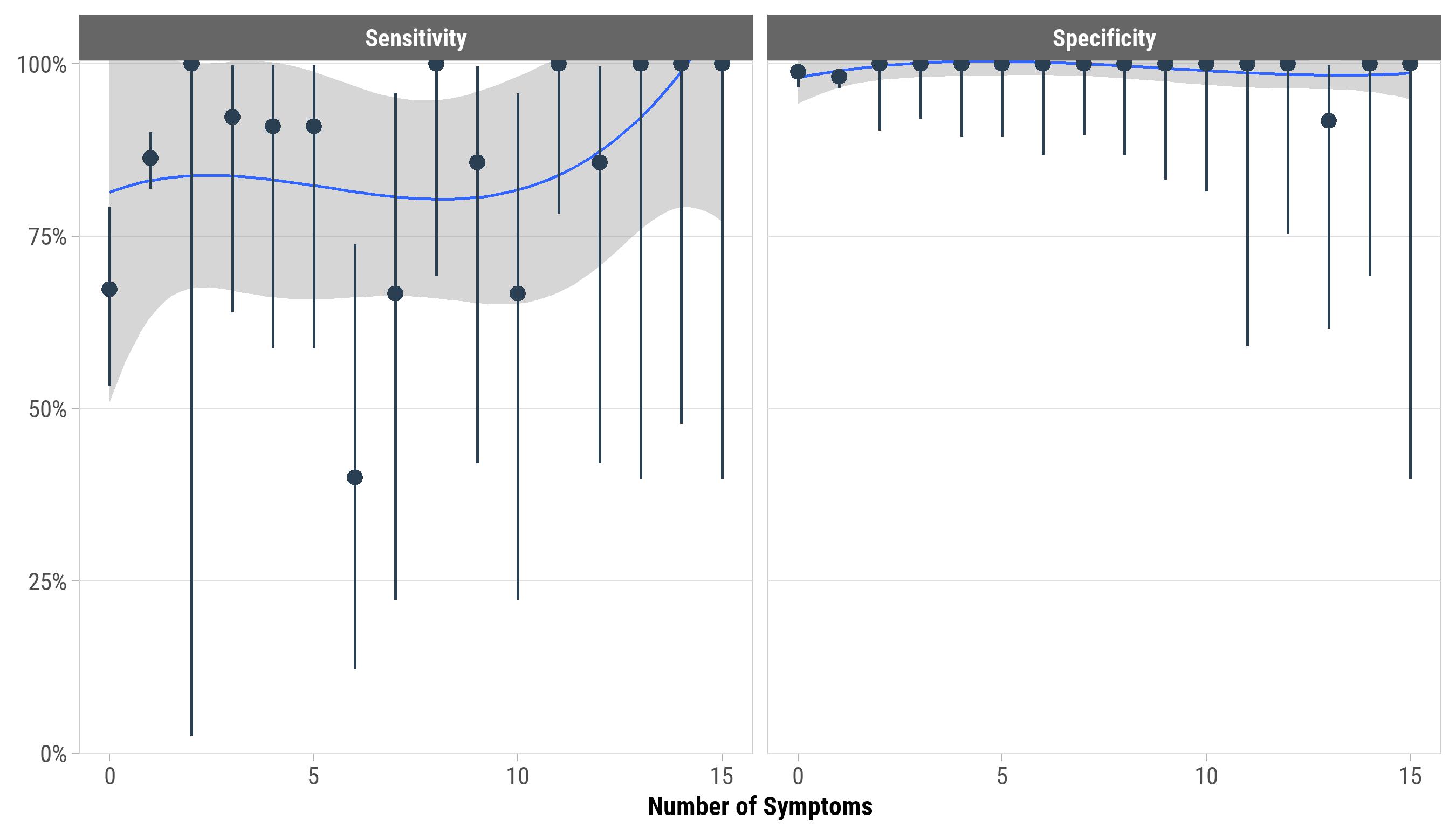


**Figure S1. Sensitivity and specificity of BinaxNOW Antigen test compared to RT-PCR by number of COVID-19 symptoms (N = 1524 paired tests from 1201 participants experiencing 0 to 16 symptoms).** The blue line represents a cubic spline and grey bands are 95% confidence intervals of the model fit. Vertical bars are 95% confidence intervals of the BinaxNOW sensitivity and specificity for each number-of-symptoms subgroup. Symptoms included tiredness, cough, loss of smell, dyspnea, myalgia, throat pain, chest pain, nausea/vomiting, diarrhea, abdominal pain, nasal congestion, chills, conjunctivitis, skin changes, rash, arthralgia, eye pain, bleeding, irritability, and calf pain. There were 1524 tests of both BinaxNOW and RT-PCR.

**
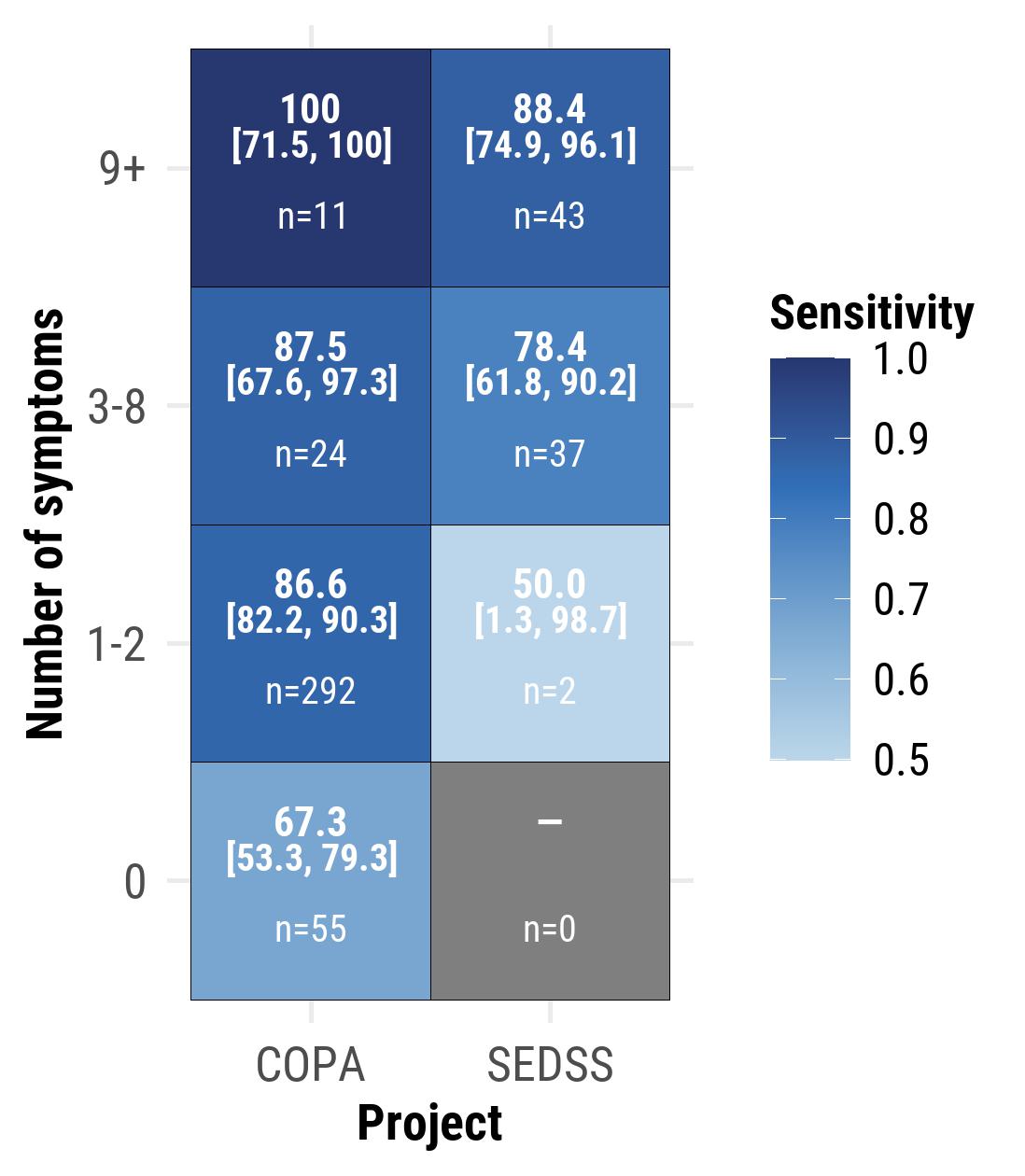
**

**Figure S2. Sensitivity of BinaxNOW Antigen test compared to RT-PCR by project (COPA, SEDSS) and number of symptoms reported (N = 1526 paired tests from 1203 participants).** Sensitivity, 95% confidence intervals, and the number of positive RT-PCR tests for each group are shown. There were 1526 tests of both BinaxNOW and RT-PCR. Symptoms included tiredness, cough, loss of smell, dyspnea, myalgia, throat pain, chest pain, nausea/vomiting, diarrhea, abdominal pain, nasal congestion, chills, conjunctivitis, skin changes, rash, arthralgia, eye pain, bleeding, irritability, and calf pain.

| Table S1. Definitions of Diagnostic Performance Metrics | |
| --- | --- |
| Term | **Definition** |
| Sensitivity | The proportion of true positive BinaxNOW tests (correctly identified infections) compared to RT-PCR as the reference standard. |
| Specificity | The proportion of true negative BinaxNOW tests (correctly identified non-infections) compared to RT-PCR as the reference standard. |
| Positive predictive value | The probability that a positive BinaxNOW result correctly indicates an actual SARS-CoV-2 infection, as determined by RT-PCR. |
| Negative predictive value | The probability that a negative BinaxNOW result correctly indicates the absence of a SARS-CoV-2 infection, as determined by RT-PCR. |
| Positive likelihood ratio | The likelihood of obtaining a positive BinaxNOW result for SARS-CoV-2 infection compared to RT-PCR, indicating diagnostic performance. |
| Negative likelihood ratio | The likelihood of obtaining a negative BinaxNOW result for SARS-CoV-2 infection compared to RT-PCR, indicating diagnostic performance. |
| Correctly classified proportion | The proportion of participants correctly classified as either SARS-CoV-2 positive or negative by BinaxNOW compared to RT-PCR results. |
| Apparent positivity | The proportion of positive BinaxNOW results in the study population, which includes both correct and incorrect diagnoses by the test. |
| True positivity | The actual proportion of SARS-CoV-2 positive individuals, determined by RT-PCR, regardless of BinaxNOW results. |
| Number needed to diagnose | The number of patients who need to be tested with BinaxNOW to correctly diagnose one person with a SARS-CoV-2 infection, as determined by RT-PCR, in the study population. |

| Table S2. Comparison of BinaxNOW and RT-PCR for initial tests and repeated tests 7–14 days later by symptom status for the initial and repeated tests (N = 368 paired^a^ tests from 184 participants). | | | | | | | | | |
| --- | --- | --- | --- | --- | --- | --- | --- | --- | --- |
|  | **True Positive,**  **n (%)** | **True Negative,**  **n (%)** | **False Positive,**  **n (%)** | **False Negative,**  **n (%)** | **Sensitivity**  **% (95% CI)** | **Specificity**  **% (95% CI)** | **Positive Predictive Value**  **% (95% CI)** | **Negative Predictive Value**  **% (95% CI)** | **McNemar’s Chi Square *P* value** |
| Symptomatic to Symptomatic | | | | | | | | | |
| First | 17 (37.8) | 26 (57.8) | 1 (2.2) | 1 (2.2) | 94.4 (72.7, 99.9) | 96.3 (81.0, 99.9) | 94.4 (72.7, 99.9) | 96.3 (81.0, 99.9) | 1 |
| Second | 9 (20.0) | 31 (68.9) | 0 (0) | 5 (11.1) | 64.3 (35.1, 87.2) | 100 (88.8, 100) | 100 (66.4, 100) | 86.1 (70.5, 95.3) | 0.074 |
| Symptomatic to Asymptomatic | | | | | | | | | |
| First | 78 (85.7) | 10 (11.0) | 2 (2.2) | 1 (1.1) | 98.7 (93.1, 100) | 83.3 (51.6, 97.9) | 97.5 (91.3, 99.7) | 90.9 (58.7, 99.8) | 1 |
| Second | 6 (6.6) | 63 (69.2) | 2 (2.2) | 20 (22.0) | 23.1 (9.0, 43.6) | 96.9 (89.3, 99.6) | 75.0 (34.9, 96.8) | 75.9 (65.3, 84.6) | <0.001 |
| Asymptomatic to Symptomatic | | | | | | | | | |
| First | 1 (5.6) | 16 (88.9) | 0 (0) | 1 (5.6) | 50.0 (1.3, 98.7) | 100 (79.4, 100) | 100 (2.5, 100) | 94.1 (71.3, 99.9) | 1 |
| Second | 9 (50.0) | 9 (50.0) | 0 (0) | 0 (0) | 100 (66.4, 100) | 100 (66.4, 100.0) | 100 (66.4, 100) | 100 (66.4, 100) | 1 |
| Asymptomatic to Asymptomatic | | | | | | | | | |
| First | 5 (16.7) | 19 (63.3) | 1 (3.3) | 5 (16.7) | 50.0 (18.7, 81.3) | 95.0 (75.1, 99.9) | 83.3 (35.9, 99.6) | 79.2 (57.8, 92.9) | 0.221 |
| Second | 1 (5.6) | 16 (88.9) | 0 (0) | 1 (5.6) | 50.0 (1.3, 98.7) | 100 (79.4, 100) | 100 (2.5, 100) | 94.1 (71.3, 99.9) | 1 |
| RT-PCR: reverse transcription polymerase chain reaction; CI: confidence interval  ^a^ There were 368 tests of both BinaxNOW and RT-PCR. | | | | | | | | | |
